# Retinoic Acid Receptor-Related Receptor Alpha Ameliorates Autoimmune Arthritis via Inhibiting of Th17 Cells and Osteoclastogenesis

**DOI:** 10.3389/fimmu.2019.02270

**Published:** 2019-10-04

**Authors:** Jin-Sil Park, Su-Jin Moon, Mi-Ae Lim, Jae-Kyeong Byun, Sun-Hee Hwang, SeungCheon Yang, Eun-Kyung Kim, Hohyun Lee, Sung-Min Kim, Jennifer Lee, Seung-Ki Kwok, Jun-Ki Min, Mi-Ock Lee, Dong-Yun Shin, Sung-Hwan Park, Mi-La Cho

**Affiliations:** ^1^The Rheumatism Research Center, Catholic Research Institute of Medical Science, College of Medicine, The Catholic University of Korea, Seoul, South Korea; ^2^Division of Rheumatology, Department of Internal Medicine, College of Medicine, Seoul St. Mary's Hospital, The Catholic University of Korea, Seoul, South Korea; ^3^College of Pharmacy, Gachon University, Incheon, South Korea; ^4^College of Pharmacy and Bio-MAX Institute, Research Institute of Pharmaceutical Sciences, Seoul National University, Seoul, South Korea; ^5^Department of Medical Life Science, College of Medicine, The Catholic University of Korea, Seoul, South Korea; ^6^Department of Biomedicine and Health Sciences, College of Medicine, The Catholic University of Korea, Seoul, South Korea

**Keywords:** rheumatoid arthritis, RORα, IL-17-producing T cells, regulatory T cells, osteoclastogenesis

## Abstract

Rheumatoid arthritis (RA) is a chronic inflammatory polyarthritis characterized by progressive joint destruction. IL-17-producing CD4^+^ T (Th17) cells play pivotal roles in RA development and progression. Retinoic acid receptor-related orphan receptor alpha (RORα) is a negative regulator of inflammatory responses, whereas RORγt, another member of the ROR family, is a Th17 lineage-specific transcription factor. Here, we investigated the immunoregulatory potential of RORα in collagen-induced arthritis (CIA) mice, an experimental model of RA. Cholesterol sulfate (CS) or SR1078, a ligand of RORα, inhibited RORγt expression and Th17 differentiation *in vitro*. In addition, fortification of RORα in T cells inhibited the expression levels of glycolysis-associated genes. We found that RORα overexpression in CIA mice attenuated the clinical and histological severities of inflammatory arthritis. The anti-arthritic effect of RORα was associated with suppressed Th17 differentiation and attenuated mTOR-STAT3 signaling in T cells. Furthermore, altered RORα activity could directly affect osteoclastogenesis implicated in progressive bone destruction in human RA. Our findings defined a critical role of RORα in the pathogenesis of RA. These data suggest that RORα may have novel therapeutic uses in the treatment of RA.

## Introduction

Rheumatoid arthritis (RA) is a progressive autoimmune polyarthritis characterized by hyperplastic synovial membrane and subsequent structural damage in affected joints. It leads to significant deterioration of the quality of life of RA patients. Although the pathogenesis of RA is not fully understood, CD4^+^ T cells have been shown to play critical roles in the development and progression of RA. Among the effector T cells, interleukin-17 (IL-17)-producing T (Th17) cells can be distinguished from Th1 or Th2 cells based on their selective expression of IL-17A, IL-17F, and IL-21 (1). Th17 cells are involved in the pathogenesis of RA with regard to synovial hypertrophy, enhanced osteoclastogenesis, and neoangiogenesis (2–4). It has been shown that hypoxia-inducible factor (HIF)-1α is an essential sensor for regulation of Th17 cell differentiation through activation of receptor-related orphan receptor (ROR)γt and IL-17A, and mTOR is required for HIF-1α signaling-induced Th17 development (5, 6). On the other hand, CD4^+^CD25^+^ regulatory T (Treg) cells are another T cell subset that mediates immune tolerance and control excessive inflammatory responses (7–9). There is accumulating evidence that the percentage of circulating Treg cells in patients with active RA is reduced compared to that in healthy controls or those with inactive RA (10). The pathophysiological importance of Th17/Treg cell imbalance has attracted interest as a new target in the treatment of RA. The reciprocal regulation of Th17/Treg imbalance can be targeted to satisfy the unmet needs for treatment-resistant RA patients, including patients who are currently using biologics, namely, genetically engineered proteins derived from human genes.

The Retinoic acid receptor-related orphan receptor alpha (RORα), also known as nuclear receptor subfamily 1 group F member 1, is a member of the steroid/thyroid hormone receptor superfamily of nuclear receptor type transcriptional factors. RORα is a potent regulator of a number of genes associated with development of the central nervous system and atherosclerosis (11, 12). A natural mutant mouse strain called *staggerer* (RORα^sg/*sg*^) has been shown to have a deletion within the RORα gene. RORα^sg/*sg*^ mice have been reported to show tremor, body imbalance, hypo-α-lipoproteinemia, and decreased serum cholesterol levels and die within 3–4 weeks after birth (13–15). Recently, RORα has been shown to be able to regulate inflammation (16). Lipopolysaccharide-stimulated macrophages from *staggerer* mice have enhanced susceptibility for the production of tumor necrosis factor-alpha (TNF-α) and IL-1β, suggesting that RORα may function as a negative regulator of inflammatory responses (17, 18).

RORγt, another orphan nuclear receptor, has been shown to be selectively expressed in Th17 cells as a Th17-specific transcription factor (19). Interestingly, RORα is also expressed in Th17 cells and induced by transforming growth factor (TGF)-β and IL-6 (20). Overexpression of RORα can directly promote Th17 differentiation (20). Furthermore, RORα and RORγt can synergistically lead to greater Th17 differentiation and cytokine expression (20). These findings suggest that RORα may have pathological roles in Th17-induced autoimmune diseases, including RA. However, there is accumulating evidence that RORα mediates anti-inflammatory responses in inflammatory diseases, such as sepsis and atherosclerosis (15, 21).

Therefore, the present study was performed to determine the effects of RORα on the development of autoimmune arthritis using a murine model of RA. To clarify the underlying mechanisms by which RORα may exert a therapeutic effect, the changes in Th17 cell and Treg cell development were determined both *in vitro* and *in vivo*. Here, we showed that cholesterol sulfate (CS) or SR1078 as a ligand of RORα could inhibit the number of Th17 cells and IL-17 production by altering transcriptional checkpoints in naïve T cells. Furthermore, we investigated whether altered RORα activity could directly affect osteoclastogenesis implicated in progressive bone destruction in human RA.

## Materials and Methods

### Animals

Four- to six-week-old male C57BL/6 and DBA/1J mice were purchased from Orient Bio Inc. (Seongnam, South Korea). Mice harboring a deletion within the RORα gene were obtained from the Jackson Laboratory (Bar Harbor, ME). These mice were maintained under specific pathogen-free conditions at the Catholic Research Institute of Medical Science, Catholic University of Korea. Animals were fed standard mouse chow and water *ad libitum*. All experimental procedures were examined and approved by the Animal Research Ethics Committee of the Catholic University of Korea, and conformed to the National Institutes of Health (USA) guidelines (permit number: 2014-0126-01, 2017-0139-03).

### CIA Induction

To induce CIA in DBA/1J mice, chicken type II collagen (CII) was dissolved overnight in 0.1 N acetic acid (4 mg/mL) with gentle rotation at 4°C. DBA/1J mice were injected intradermally at the base of the tail with 100 μg of CII emulsified in Freund's adjuvant (Chondrex, Redmond, WA). Two weeks later, 100 μg of CII dissolved and emulsified at 1:1 with incomplete Freund's adjuvant (Difco, Detroit, MI) was administered to the hind legs of mice as a booster injection. To assess the effects of SR1078 on the severity of CIA, DBA/1J mice were treated with 10 mg/kg SR1078 in saline or with vehicle alone via intraperitoneal injections three times per week for 8 weeks on day 7 or 19 after the 1st immunization. For administration of pcDNA–RORα, on day 8 after the 1st immunization, DBA/1J mice were injected intravenously with 100 μg of pcDNA–RORα, or with mock vector as a control in 2 mL of saline within 5 s. Eight days after hydrodynamic intravenous injection, the same mice received intramuscular injection by electroporation of 100 μg of pcDNA–RORα or mock vector into the left leg. Intramuscular injection was performed using a 31-gauge needle insulin syringe. Seven days later, mice were injected intramuscularly with 100 μg of pcDNA–RORα in the right leg with electroporation (22, 23).

### Clinical Assessment of Arthritis

The severity of arthritis was determined by three independent observers. The mice were observed twice a week for the onset and severity of joint inflammation for up to 8 weeks after the primary immunization. The severity of arthritis was assessed on a scale of 0–4 with the following criteria, as described previously (24): 0 = no edema or swelling; 1 = slight edema and erythema limited to the foot or ankle; 2 = slight edema and erythema from the ankle to the tarsal bone; 3 = moderate edema and erythema from the ankle to the tarsal bone; and 4 = edema and erythema from the ankle to the entire leg. The arthritis score for each mouse was expressed as the sum of the scores of all four limbs. The highest possible arthritis score for a mouse was therefore 16. The mean arthritis index was used to compare the data among the control and experimental groups.

### Histological Evaluation and Immunohistochemistry

Joint tissues were fixed in 10% (v/v) neutral buffered formalin, decalcified in a histological decalcifying agent (Calci-Clear Rapid; National Diagnostics, Atlanta, GA), embedded in paraffin, and cut into sections 5 μm thick. The sections were stained with hematoxylin and eosin (H&E) and Safranin O to detect proteoglycans. Inflammation was scored using the following criteria: 0 = no inflammation; 1 = slight thickening of the lining or infiltration of some cells into the underlying layer; 2 = slight thickening of the lining with infiltration of some cells into the underlying layer; 3 = thickening of the lining, with an influx of cells into the underlying layer, and cells evident in the synovial space; and 4 = extensive infiltration of the synovium by inflammatory cells. Cartilage damage was evaluated by staining with Safranin O and toluidine blue, and the extent of damage was scored using the following criteria: 0 = no destruction; 1 = minimal erosion (limited to single spots); 2 = slight-to-moderate erosion in a limited area; 3 = more extensive erosion; and 4 = general destruction. Immunohistochemistry was performed using a Vectastain ABC kit (Vector Laboratories, Burlingame, CA). Tissues were stained with anti-TNF-α, anti-IL-1β, anti-IL-6, anti-IL-17, and anti-vascular endothelial growth factor (VEGF) antibodies and an isotype control (Santa Cruz Biotechnology, Santa Cruz, CA). Cells were counted visually at higher magnification by projection on a screen and cytokine-positive cells were identified by their brown color.

### Confocal Microscopy

Spleen tissues were snap-frozen in liquid nitrogen and stored at −70°C. Spleen tissue sections (7 μm) were fixed in acetone and stained for Treg cells using fluorescein isothiocyanate (FITC)-labeled anti-Foxp3, phycoerythrin (PE)-labeled anti-CD4 (both from eBioscience, San Diego, CA), and allophycocyanin (APC)-labeled anti-CD25 (BioLegend, San Diego, CA) antibodies. To stain Th17 cells, PE-labeled anti-IL-17 (eBioscience), FITC-labeled anti-CD4 (eBioscience), and PE-labeled anti-phosphorylated STAT-3 (pTyr705 or pSer727; BD Biosciences) antibodies were used. Tissue and cells were stained at 4°C overnight with an antibody against HIF-1α (Abcam, Cambridge, UK), pmTOR (Cell Signaling Technology, Beverly, MA), and CD4 (BioLegend). LBRM-33 cells were centrifuged onto slides using CytoSpin III (Shandon Scientific, Pittsburgh, PA) at 700 rpm for 5 min. Cells were air-dried, fixed with methanol, and blocked with 10% goat serum at room temperature for 30 min. After incubation with appropriate staining antibodies at 4°C overnight, sections were analyzed by confocal microscopy (LSM 510 Meta; Carl Zeiss, Oberkochen, Germany). Positive cells were counted visually at higher magnification by four individuals.

### Enzyme-Linked Immunosorbent Assay (ELISA)

The amounts of IL-17 and TNF-α in culture supernatants from mouse or human cells were measured by sandwich ELISA (R&D Systems, Minneapolis, MN). Horseradish peroxidase–avidin (R&D Systems) was used for color development. Blood from the orbital sinus of mice was taken and serum samples were stored at −20°C until use. The levels of immunoglobulin (Ig)G, IgG1, and IgG2a were measured using a mouse ELISA quantification kit (Bethyl Lab Co., Montgomery, TX). The absorbance was determined at a wavelength of 405 nm on an ELISA microplate reader (Molecular Devices, Sunnyvale, CA).

### Isolation of Splenocytes and CD4^+^ T Cells

Isolation of mouse splenocytes and splenic CD4^+^ T cells and differentiation of effector T cells were performed as described previously (22). To purify splenic CD4^+^ T cells, the splenocytes of mice were incubated with CD4-coated magnetic beads and isolated using magnetic activated cell sorting separation columns (Miltenyi Biotech, Auburn, CA). To establish Th17 cell-polarizing conditions, the sorted CD4^+^ T cells were stimulated with plate-bound anti-CD3 (0.5 μg/mL), anti-CD28 (1 μg/mL), anti-interferon (IFN)-γ (2 μg/mL), anti-IL-4 (2 μg/mL), TGF-β (2 ng/mL), and IL-6 (20 ng/mL) for 72 h. Cells were pretreated with CS (Sigma, St. Louis, MO) for 1 day and then stimulated under the appropriate polarizing conditions.

### Flow Cytometry

Expression levels of cytokines and transcription factors were assessed by intracellular staining using the following antibodies. For intracellular staining: anti-IL-17-FITC, anti-Foxp3-FITC, and anti-Foxp3-PE (all from eBioscience). Cells were stimulated with PMA and ionomycin with the addition of GolgiStop for 4 h. Cultured cells were surface labeled for 30 min and permeabilized with Cytofix/Cytoperm solution (BD Pharmingen, Heidelberg, Germany). Cells were intracellularly stained with fluorescent antibodies before flow cytometry (FACSCalibur; BD Biosciences, Franklin Lakes, NJ). Events were collected and analyzed with FlowJo software (Tree Star, Ashland, OR).

### Western Blotting

Cells were lysed in Halt protein lysis buffer containing Halt phosphatase inhibitor (Thermo Pierce, Waltham, MA). Lysates were centrifuged at 14,000 × *g* for 15 min at 4°C. Protein concentration was determined by Bradford protein assay (Bio-Rad, Hercules, CA). Proteins were separated by SDS-PAGE and transferred onto Hybond ECL membranes (GE Healthcare, Waukesha, WI) for Western blotting analysis using SNAP i.d. Protein Detection System (Millipore, Billerica, MA). Blots were incubated with antibodies against RORα (Santa Cruz) and β-actin (Sigma). After washing, HRP-conjugated secondary antibodies were added. Hybridized bands were detected using an ECL detection kit (Pierce, Rockford, IL) and Hyperfilm (Agfa, Mortsel, Belgium).

### Gene Expression Analysis Using Real-Time Polymerase Chain Reaction

A LightCycler 2.0 instrument (software version 4.0; Roche Diagnostics, Penzberg, Germany) was used for PCR amplification. All reactions were performed with LightCycler FastStart DNA Master SYBR Green I (Takara, Kyoto, Japan) according to the manufacturer's instructions. The following primers were used: RORα, 5′-GGAAGGTCTGCCACGTTATCTG−3′ (sense) and 5′-TCCAAATCCCACCTGGAAAC−3′ (antisense); RORγT, 5′-TGTCCTGGGCTACCCTACTG−3′ (sense) and 5′-GTGCAGGAGTAGGCCACATT−3′ (antisense); IL-17A, 5′-CCTCAAAGCTCAGCGTGTCC−3′ (sense) and 5′-GAGCTCACTTTTGCGCCAAG−3′ (antisense); Foxp3, 5′-GGCCCTTCTCCAGGACAGA−3′ (sense) and 5′-GCTGATCATGGCTGGGTTGT−3′ (antisense); STAT3, 5′-CCGTCTGGAAAACTGGATAACTTC−3′ (sense) and 5′-CCTTGTAGGACACTTTCTGCTGC−3′ (antisense); HIF-1α, 5′-AGGCCTAGATGGCTTTGTGA−3′ (sense) and 5′-TATCGAGGCTGTGTCGACTG−3′ (antisense); Glut1, 5′-CAGTTCGGCTATAACACTGGTG−3′ (sense) and 5′-GCCCCCGACAGAGAAGATG−3′ (antisense); MCT4, 5′-TCACGGGTTTCTCCTACGC−3′ (sense) and 5′-GCCAAAGCGGTTCACACAC−3′ (antisense); HK2, 5′-TGATCGCCTGCTTATTCACGG−3′ (sense) and 5′- AACCGCCTAGAAATCTCCAGA−3′ (antisense); GPI, 5′-TCAAGCTGCGCGAACTTTTTG−3′ (sense) and 5′- GTTCTTGGAGTAGTCCACCAG−3′ (antisense); TPI, 5′-CCAGGAAGTTCTTCGTTGGGG−3′ (sense) and 5′-CAAAGTCGATGTAAGCGGTGG−3′ (antisense); Eno1, 5′-TGCGTCCACTGGCATCTAC−3′ (sense) and 5′-CAGAGCAGGCGCAATAGTTTTA−3′ (antisense); PKM, 5′-GCCGCCTGGACATTGACTC−3′ (sense) and 5′-CCATGAGAGAAATTCAGCCGAG−3′ (antisense); LDHα, 5′-CATTGTCAAGTACAGTCCACACT−3′ (sense) and 5′- TTCCAATTACTCGGTTTTTGGGA−3′ (antisense); TRAP, 5′-TCCTGGCTCAAAAAGCAGTT−3′ (sense) and 5′-ACATAGCCCACACCGTTCTC−3′ (antisense); cathepsin K, 5′-CAGCAGAGGTGTGTACTATG−3′ (sense) and 5′-GCGTTGTTCTTATTCCGAGC−3′ (antisense); calcitonin receptor, 5′-CGGACTTTGACACAGCAGAA−3′ (sense) and 5′-AGCAGCAATCGACAAGGAGT−3′ (antisense); p53, 5′-CACGTACTCTCCTCCCCTCA−3′ (sense) and 5′-CTCCGTCATGTGCTGTGACT−3′ (antisense); and β-actin, 5′-GTACGACCAGAGGCATACAGG−3′ (sense) and 5′-GATGACGATATCGCTGCGCTG−3′ (antisense). The level of mRNA expression was normalized to that of β-actin mRNA.

### *In vitro* Osteoclastogenesis and Tartrate-Resistant Acid Phosphatase (TRAP) Staining

Isolation of mouse bone marrow cells, differentiation of osteoclasts, and TRAP staining were performed as described previously (25).

### Cell Viability Analysis

Cell viability was determined using a CCK-8 kit (Dojindo Laboratories, Kumamoto, Japan) according to the manufacturer's instructions. Briefly, splenic CD4^+^ T cells (2 × 10^5^ cells/well, 96-well plate) were pre-stimulated with CS for 1 day and cultured with anti-CD3 and anti-CD28 for 3 days. CCK-8 (10 μL) was added to each well of the plate. After incubation for 3 h, the absorbance at 450 nm of each well was measured on a microplate reader.

### Statistical Analysis

Statistical analyses were performed using SAS software (version 9.2; SAS Institute, Cary, NC). Normally distributed continuous data were analyzed using the parametric Student's *t*-test. Non-normally distributed data were analyzed using the non-parametric Mann–Whitney *U*-test. Differences in mean values of various groups were analyzed by analysis of variance (ANOVA) with a *post hoc* test. Experimental values are presented as means ± SD. In all analyses, *P* < 0.05 (2-tailed) was taken to indicate statistical significance.

## Results

### RORα Is Capable of Suppressing Th17 Cell Differentiation *in vitro*

**Figure 1:**
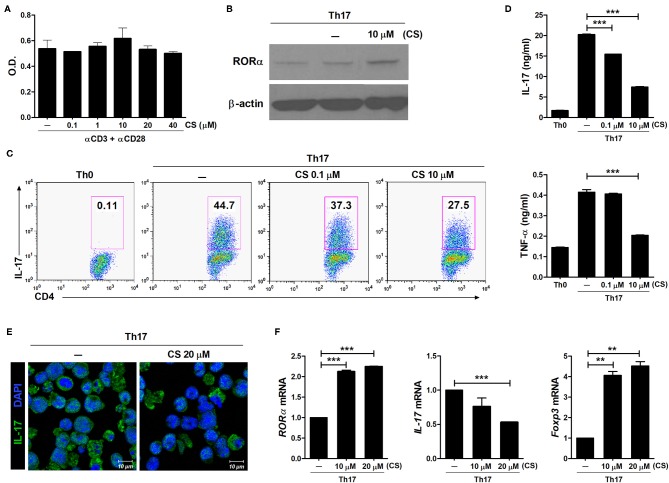
RORα suppresses Th17 cell differentiation *in vitro*. **(A)** Splenic CD4^+^ T cells from normal 6-week-old C57BL/6 mice were pretreated with CS, a ligand for RORα, for 24 h and then stimulated with anti-CD3/anti-CD28 monoclonal antibodies for 72 h. Cell viability was measured using the CCK-8 method. **(B)** Immunoblotting analysis was performed to determine the expression level of RORα in CS-treated CD4^+^ T cells. Cropped blots are displayed. **(C)** Murine CD4^+^ T cells of C57BL/6 mice were cultured under Th17 cell-polarizing conditions with or without various concentrations of CS for 72 h. These cells were then stained with anti-CD4 and anti-IL-17 antibodies for intracellular flow cytometry analysis. Representative results are shown. **(D)** Concentrations of IL-17 and TNF-α in culture supernatants of CS-stimulated T cells in **(C)** were measured by ELISA. **(E)** CS treatment inhibited IL-17 expression in LBRM-33 cells stimulated under Th17-polarizing conditions. The expression of IL-17 was analyzed by immunostaining and confocal microscopy (×800). **(F)** Expression levels of RORα, IL-17, and Foxp3 in CS-stimulated LBRM-33 cells were determined by real-time polymerase chain reaction. Data are presented as means ± SD of two independent experiments. **P < 0.01, ***P < 0.001.

CS is known to induce RORα transcriptional activity (26–28). To determine whether RORα overexpression could regulate the development of Th17 cells, murine T cells were cultured in the presence of CS, a putative natural ligand of RORα (29). First, murine splenic CD4^+^ T cells were stimulated with anti-CD3 and anti-CD28 antibodies in the presence of CS. CS at concentrations from 0.1 to 40 μM showed no cellular toxicity to murine T cells in culture for 72 h (Figure 1A). To confirm increased RORα expression, murine splenic CD4^+^ T cells were pretreated for 24 h with CS and then cultured for an additional 24 h under Th17-polarizing conditions. RORα expression was found to be higher following CS treatment compared to that in untreated cells (Figure 1B). Next, we examined the effects of CS on Th17 cell differentiation *in vitro*. Murine CD4^+^ T cells were cultured in the presence of anti-CD3, anti-CD28, anti-IFN-γ, anti-IL-4 antibodies, TGF-β, and IL-6 with or without CS for 72 h. After stimulation under conditions favoring Th17 cell differentiation, flow cytometry indicated that CS-stimulated T cells were less prone to differentiate toward Th17 cells compared to untreated cells (Figure 1C). The amounts of IL-17A and TNF-α in culture supernatants of CS-treated T cells were significantly (*P* < 0.05) lower than those in culture supernatants of untreated cells (Figure 1D). Next, LBRM-33 murine T lymphoma cells were stimulated with CS for 24 h and then cultured under Th17-polarizing conditions for an additional 48 h. Overexpression of RORα by CS resulted in significantly (*P* < 0.05) attenuated IL-17 expression in LBRM-33 cells, whereas Foxp3 expression was reciprocally and significantly (*P* < 0.05) increased (Figures 1E,F). Taken together, these results suggest that inducing RORα activity in T cells may represent a novel treatment strategy for management of various Th17-associated diseases, including RA.

### Fortification of RORα Using Various Inducers Inhibits IL-17 Production

**Figure 2:**
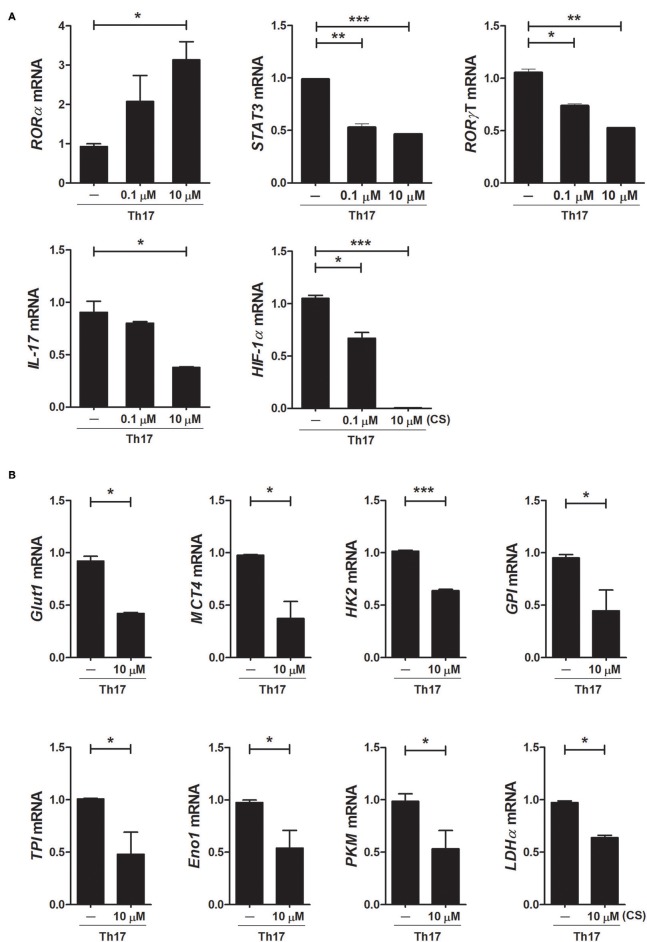
Overexpression of RORα alters the expression levels of metabolic genes in Th17 cells. **(A)** CD4^+^ T cells of normal C57BL/6 mice were pretreated with various concentrations of CS and then cultured under Th17-skewing conditions for 72 h. The expression levels of RORα, RORγt, STAT3, IL-17, and HIF-1α in these cells were determined by real-time PCR. **(B)** CD4^+^ T cells of normal C57BL/6 mice were pretreated with various concentrations of CS and then cultured under Th17-skewing conditions for 72 h. The expression levels of glycolysis-associated molecules were determined by real-time PCR. Data are presented as means ± SD of two independent experiments. *P < 0.05, **P < 0.01, ***P < 0.001.

**Figure 3:**
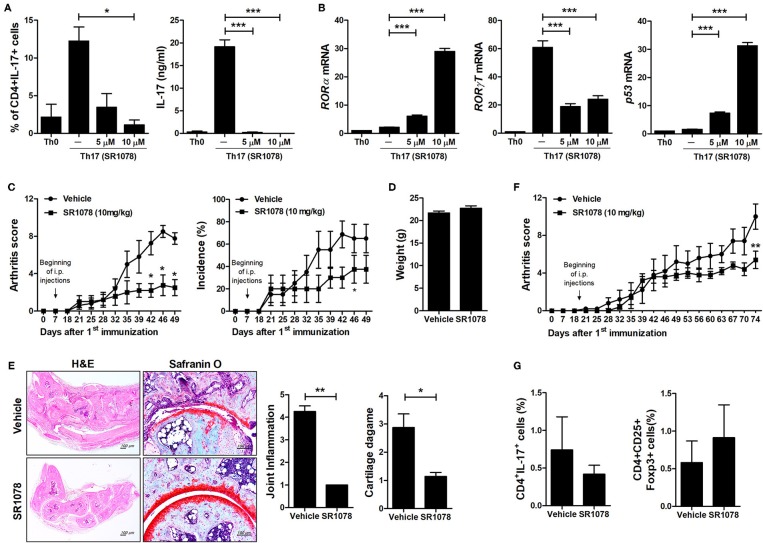
SR1078 inhibits the production of IL-17 and the development of arthritis. **(A,B)** CD4^+^ T cells of normal C57BL/6 mice were pretreated with various concentrations of SR1078 and then cultured under Th17-skewing conditions for 72 h. **(A)** These cells were then stained with anti-CD4 and anti-IL-17 antibodies for intracellular flow cytometry analysis. The level of IL-17 in the culture supernatant was measured by ELISA. **(B)** The expression levels of RORα, RORγt, and p53 in these cells were determined by real-time PCR. **(C–E)** At 7 days after 1st immunization, mice were treated with 10 mg/kg SR1078 in saline or with vehicle alone via intraperitoneal injections three times per week (*n* = 4/group). Arthritis development was assessed using the arthritis score (left) and incidence (right). **(D)** The graph shows body weight at 49 days after the first immunization. **(E)** Sections of articular tissue were prepared from mice treated as described in **(C)** 49 days after the first immunization and stained with H&E and safranin O. Representative histological features are shown. The graphs depict the degree of inflammation, bone damage, and cartilage damage. **(F,G)** At 19 days after the 1st immunization, mice were treated with 10 mg/kg SR1078 in saline or with vehicle alone via intraperitoneal injections three times per week (*n* = 5/group). Clinical score of arthritis is shown for each treatment group over time. **(G)** At 74 days after the first immunization, the number of CD4 + IL-17 + or CD4 + CD25 + Foxp3 + cells in *ex vivo* splenocytes was analyzed by flow cytometry. *P < 0.05, **P < 0.01, ***P < 0.001 vs. vehicle-treated mice.

To investigate the mechanisms underlying the inhibition of Th17 differentiation by CS, the expression levels of Th17-related mediators were examined. STAT3 is an essential transcription factor of Th17 cell differentiation through RORγt induction by binding to sites within the first intron of *RORc*, which encodes RORγt (19, 30, 31). As expected, CS treatment induced RORα mRNA expression in murine T cells but significantly suppressed STAT3, RORγt, and IL-17 mRNA expression (Figure 2A). Recent studies indicated that HIF-1α can induce Th17 development through directly activating the transcription of RORγt while reciprocally attenuating Treg development by directly targeting Foxp3 (5, 6). Our study indicated that overexpression of RORα in T cells suppressed HIF-1α mRNA expression (Figure 2A). Next, we examined whether changes caused by CS treatment in the process of HIF-1α-dependent glycolytic activity were required for suppressed Th17 cell differentiation. Real-time PCR results indicated that the expression levels of genes encoding glycolysis-associated molecules in CS-stimulated T cells cultured under Th17-skewing conditions were significantly (*P* < 0.05) downregulated compared to those in untreated cells (Figure 2B). To confirm the inhibitory effects of RORα on Th17 cell differentiation, murine T cells were cultured in the presence of anti-CD3, anti-CD28, anti-IFN-γ, anti-IL-4 antibodies, TGF-β, and IL-6 with or without SR1078, a selective RORα ligand, for 72 h (32). SR1078 treatment significantly suppressed number of Th17 cells and production of IL-17 (Figure 3A). Furthermore, SR1078 treatment increased the expression of RORα and p53 mRNA in murine T cells but significantly decreased the expression of RORγt mRNA (Figure 3B). To investigate whether chemical inducer of RORα has prophylactic activity in the progress of arthritis *in vivo*, DBA/1J mice were treated with SR1078 via intraperitoneal injections three times per week for 6 weeks on day 7 after the 1st immunization. SR1078 treatment did not affect body weight changes (Figure 3D), but arthritic severity was controlled from the beginning of arthritis development and intra-articular inflammation and cartilage damage were improved compared to the control group (Figures 3C,E). To examine the therapeutic effects of SR1078, SR1078 was injected intraperitoneally in CIA mice at 5 days after second CII immunization. SR1078 treatment in arthritis mice ameliorated the arthritis score (Figure 3F). Although there was no statistical significance, the number of Treg cells increased while the number of Th17 decreased in the SR1078 injection group compared to the control group (Figure 3G). These results suggested that RORα-induced suppression of Th17 cells may be achieved by altering complicated transcriptional checkpoints in naïve T cells and it could work on the *in vivo* system.

### RORα Modulates the Severity of Autoimmune Arthritis

**Figure 4:**
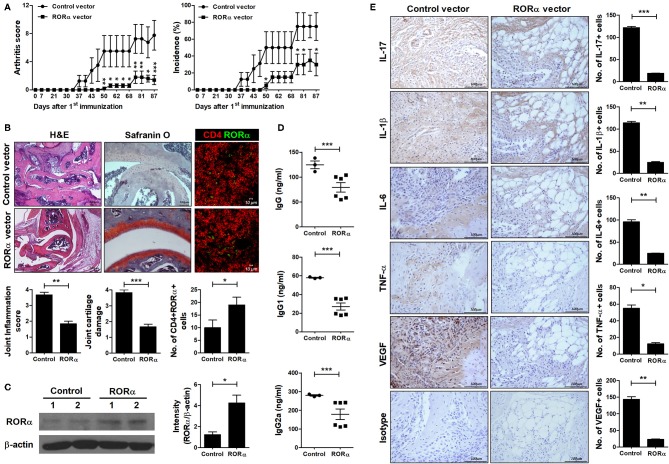
*In vivo* therapeutic effect of RORα on the development of autoimmune arthritis. Eight days after immunization with CII, mice with CII-induced arthritis (CIA) were injected intravenously with pcDNA–RORα or pcDNA mock control vector (*n* = 6 or *n* = 3/group, respectively). On days 8 and 15 after hydrodynamic intravenous injection, mice received additional injection of pcDNA–RORα or pcDNA mock control vector by electroporation into the muscles of both thighs. **(A)** Clinical scores of arthritis (left) and incidence of arthritis (right) are shown for each treatment group over time (representative results from one of two independent experiments). **(B)** At 90 days after the first CII immunization, tissue sections were obtained from the paw and ankle joints of mice with CIA and stained with hematoxylin and eosin (H&E; original magnification × 40) and Safranin O (original magnification × 200) to examine the severity of arthritis. Tissue sections were obtained from spleen of mice with CIA and immunostained to detect the level of RORα in CD4+ cells (left). The histological scores of inflammation, cartilage damage and the number of CD4^+^RORα^+^ cells were determined (right). **(C)** At 90 days after the first CII immunization, spleens were obtained from spleen of mice treated with pcDNA mock control or pcDNA-RORα vector and performed western blot to detect the level of RORα. **(D)** Concentrations of IgG, IgG1, and IgG2a in the sera of mice from each treatment group were measured by ELISA. Data are expressed as dot plots with the mean (bar) for three (pcDNA mock control vector) or six (pcDNA–RORα) animals from each group. **(E)** The expression levels of IL-17, IL-1β, IL-6, TNFα, and VEGF in the ankle joints were determined by immunohistochemical staining. Bars indicate the means ± SD of three (pcDNA mock control vector) or six (pcDNA–RORα) mice per group. *P < 0.05, **P < 0.01, ***P < 0.001 vs. pcDNA mock control vector-treated mice.

To further examine whether overexpression of RORα could modulate the development and severity of arthritis *in vivo*, pcDNA–RORα was administered to CIA mice at 8 days after CII immunization. Our results showed that level of RORα increased by administration of pcDNA–RORα (Figures 4B,C) and RORα overexpression in arthritis mice reduced the arthritis score and the incidence of arthritis compared to those in mice receiving control pcDNA vector from the early phase of the disease until 90 days after arthritis induction (Figure 4A). On histological examination of the joints, the paws and ankles of CIA mice injected with pcDNA–RORα exhibited a significantly lower degree of inflammation (*P* < 0.05) and significantly attenuated cartilage damage (*P* < 0.05) compared to those of mice treated with control vector (Figure 4B). In addition, serum levels of IgG, IgG1, and IgG2a in mice injected with pcDNA–RORα were significantly lower (*P* < 0.05) than those in mice treated with control vector (Figure 4D). IL-17, IL-1β, IL-6, and TNF-α are cytokines that have been successfully targeted in the treatment of RA in numerous clinical trials (33). These cytokines cause synovial inflammation with systemic effects (34). Excessive angiogenesis can maintain chronic inflammation by transporting inflammatory cells and supplying oxygen to inflamed joints (35). Although enhanced angiogenesis is associated with inflammatory conditions, rather than being a disease-specific phenomenon, the targeting of angiogenesis has attracted attention in RA treatment (36–38). Our results indicated that the joints of pcDNA–RORα-treated mice with CIA had markedly lower levels of IL-1β, IL-6, TNF-α, IL-17, and VEGF expression than those of mice treated with control pcDNA vector (Figure 4E). These results suggest that the overexpression of RORα can alleviate the development of inflammatory arthritis *in vivo*.

### Anti-inflammatory Properties of RORα in Mice With Autoimmune Arthritis Are Associated With Th17 Suppression

**Figure 5:**
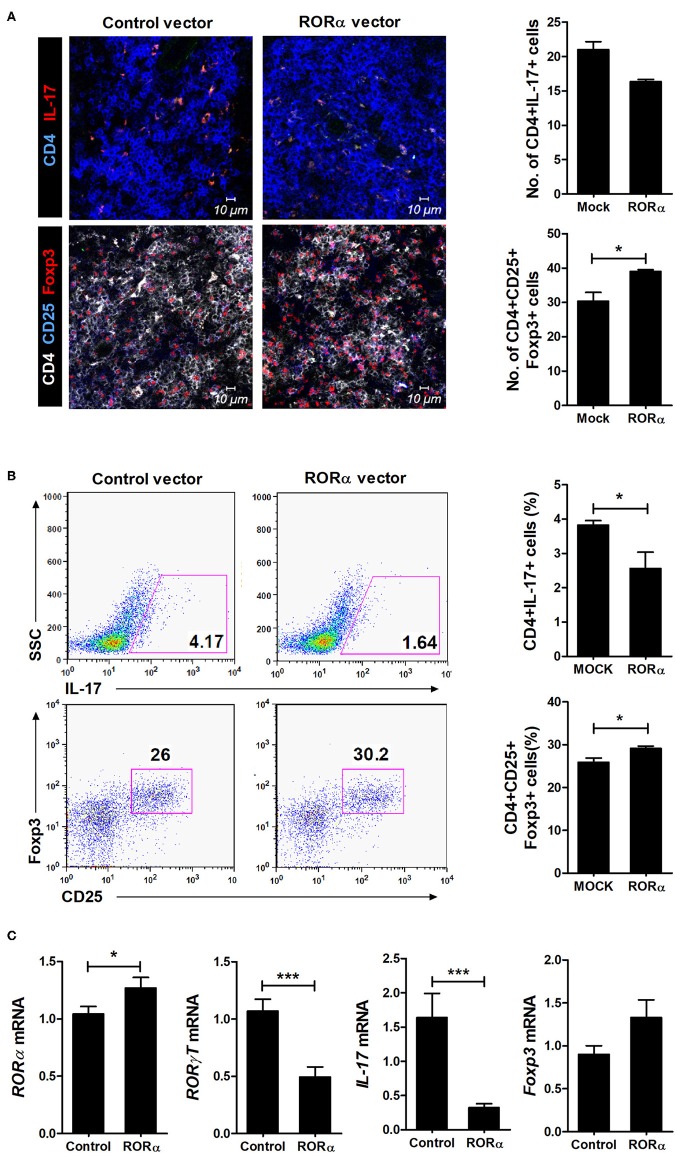
Reciprocal effects of RORα treatment on differentiation of Th17 and Treg cells *in vivo*. At 90 days after the first CII immunization, spleens were obtained from pcDNA–RORα- or pcDNA mock control vector-treated CIA mice. **(A)** Spleens were examined by immunofluorescence staining with monoclonal antibodies against CD4, IL-17, CD25, and Foxp3. Original magnification × 400. Right, CD4(blue)^+^IL-17(red)^+^ Th17 cells and CD4(white)^+^CD25(blue)^+^Foxp3(red)^+^ Treg cells were counted visually at higher magnification by projection onto a screen (with each confocal image representative of four fields of view). Results are presented as means ± SD for the number of positive cells in three mice per group. **(B)** The proportions of IL-17^+^ or CD25^+^Foxp3^+^ cells among splenic CD4^+^ T cells in each group of mice were assessed by flow cytometry. Representative flow cytometry plots from one of two independent experiments are shown (left). Results are presented as means ± SD for three mice per group (right). **(C)** Expression levels of RORα, RORγt, IL-17, and Foxp3 of isolated splenocytes described in **(B)** were determined by real-time PCR. Bars show the means ± SD results in three mice per group from at least three independent experiments. *P < 0.05, ***P < 0.001 vs. mock control vector-treated mice.

**Figure 6:**
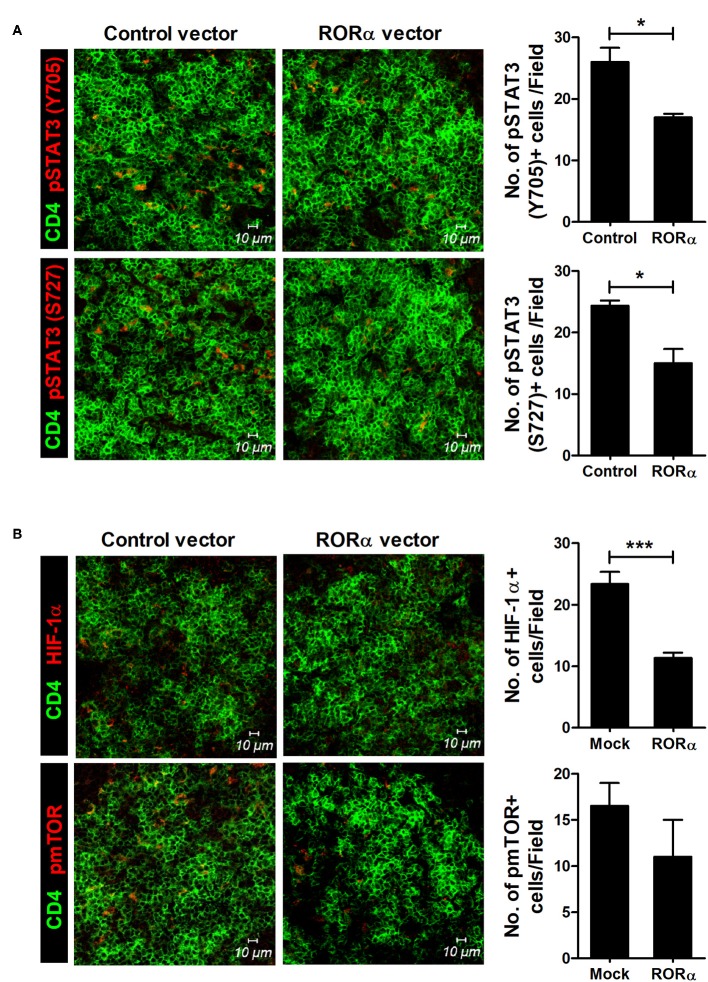
RORα modulates mTOR-STAT3 signaling pathway. At 90 days after the first CII immunization, spleens were isolated from pcDNA–RORα- or pcDNA mock control vector-treated CIA mice and subjected to immunofluorescence staining with monoclonal antibodies against CD4 (green), pSTAT3 (Tyr705; red), pSTAT3 (Ser727; red) **(A)**, HIF-1α (red), or phosphorylated mTOR (red) **(B)**. Cells were counted visually at higher magnification by projection onto a screen. Results are presented as means ± SD of three mice per group (right bar graph). *P < 0.05, ***P < 0.001 vs. pcDNA mock control vector-treated mice.

To examine whether RORα overexpression could alter the populations of Th17 and Treg cells, IL-17-expressing cells (mainly Th17) and CD25^+^Foxp3^+^ cells (mainly Treg) among CD4^+^ T cells in the spleens of arthritic mice were analyzed by confocal microscopy and flow cytometry. Our results revealed that pcDNA–RORα-treated CIA mice had a slight increase in the number of Foxp3-expressing Treg cells with a reciprocal decrease in the number of Th17 cells compared to mice treated with control vector (Figures 5A,B). In addition, the mRNA expression levels of RORα and Foxp3 were increased, whereas those of RORγt and IL-17 were decreased in splenocytes isolated from pcDNA–RORα-treated CIA mice compared to those isolated from CIA mice treated with mock vector (Figure 5C). The numbers of pSTAT3 (Y705 and S727)-expressing CD4^+^ T cells in the spleens of pcDNA–RORα-treated CIA mice were significantly (*P* < 0.05) decreased compared to those in the spleens of CIA mice treated with mock vector (Figure 6A). Confocal microscopy also revealed that the proportions of HIF-1α-expressing CD4^+^ T cells were significantly decreased in pcDNA–RORα-treated CIA mice (Figure 6B). As mTOR is required for HIF-1α signaling-induced Th17 development and diminished Treg differentiation (6), we examined whether the reciprocal regulation of Th17/Treg cells in pcDNA–RORα-treated arthritic mice was dependent on reduced HIF-1α signaling. As expected, the number of pmTOR-expressing CD4^+^ splenic T cells was decreased in CIA mice injected with pcDNA–RORα along with reduced expression of HIF-1α (Figure 6B). These results suggest that overexpression of RORα in mice with autoimmune arthritis exerts anti-inflammatory effects associated with reciprocal regulation of Th17/Treg cell populations *in vivo*.

### Inhibition of Osteoclastogenesis by RORα

**Figure 7:**
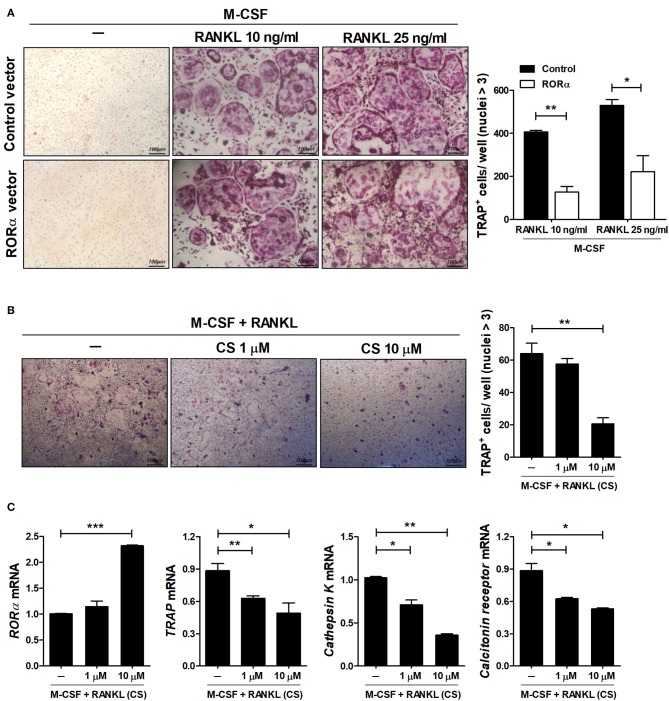
RORα inhibits osteoclastogenesis. **(A)** Osteoclast precursor cells from BMM cells of CIA mice treated with pcDNA–RORα or pcDNA control vector were further cultured in the presence of macrophage colony-stimulating factor (M-CSF) (10 ng/mL) alone or together with RANKL (10 or 25 ng/mL). After 4 days, cells were stained for TRAP (representative images are shown). The number of multinucleated TRAP^+^ cells was determined. **(B,C)** BMM cells of normal C57BL/6 mice were cultured in the presence of M-CSF and RANKL with or without CS for 4 days. The number of multinucleated TRAP^+^ cells was determined. **(C)** The mRNA expression levels of RORα, TRAP, cathepsin K, and calcitonin receptor were determined by real-time PCR. Data are presented as means ± SD of three independent experiments. *P < 0.05, **P < 0.01, ***P < 0.001.

RA is a prototypical inflammatory arthritis characterized by devastating inflammation-driven cartilage and bone destruction. Joint destruction in RA is mainly attributable to abnormal activation of osteoclasts responsible for bone resorption regulated by macrophage colony-stimulating factor (M-CSF) and receptor activator of NFκB ligand (RANKL) (39, 40). Previous reports have shown that mutant mouse *staggerer* (*sg/sg*) carrying a deletion within the RORα gene is osteopenic with thin long bones compared to wild-type mice (41). To examine the effects of RORα on *in vitro* osteoclastogenesis, bone marrow-derived monocyte/macrophage (BMM) cells isolated from pcDNA–RORα or control pcDNA vector-injected CIA mice were stimulated with M-CSF alone or together with RANKL. Based on TRAP staining, our results indicated that overexpression of RORα in arthritic mice significantly (*P* < 0.05) reduced osteoclast differentiation (Figure 7A). To investigate the direct effects of RORα activity on *in vitro* osteoclastogenesis, BMM cells were cultured with M-CSF and RANKL in the presence or absence of CS. CS treatment in murine BMM cells effectively inhibited the differentiation of osteoclasts in a dose-dependent manner (Figure 7B). To characterize the molecular mechanisms involved in the attenuation of osteoclastogenesis in CS-treated BMM cells, mRNA levels of various osteoclastogenic markers, such as TRAP, cathepsin K, and calcitonin receptor, were measured by real-time PCR. The mRNA expression levels of all of these osteoclastogenic markers were decreased by CS in a dose-dependent manner (Figure 7C). These results suggested that RORα may have novel therapeutic uses for inhibition of the progression of joint destruction in RA patients, which is mainly caused by activated osteoclasts.

## Discussion

In this study, we investigated whether RORα activity could modulate inflammatory responses in a murine model of RA. Our results indicated that overexpression of RORα *in vivo* significantly reduced the clinical and histological severities of autoimmune arthritis. Enhanced RORα activity exerted its anti-inflammatory effects by suppressing the differentiation of Th17 cells. Interestingly, CS-induced suppression of Th17 cell differentiation was associated with inhibition of HIF-1α and decreased expression of glycolysis-associated molecules. It is increasingly evident that metabolic reprogramming has a potential role in the control of cellular differentiation, activation, and function. However, the fundamental mechanisms that mediate such processes remain to be elucidated. Our results suggest that enhancing RORα activity in T cells under an inflammatory milieu where naïve CD4^+^ T cells can be differentiated into Th17 cells may be useful as a novel treatment strategy for Th17-associated diseases, such as RA.

Retinoic-related orphan receptors RORα, β, and γ constitute a subfamily of nuclear receptors that can regulate gene transcription by binding to ROR-response elements in the promoters of target genes as a monomer (12, 42, 43). RORα as a constitutive activator of transcription can modulate a wide spectrum of genes expressed in various organs, including the brain, skeletal muscle, kidney, and hair follicles (42, 44). It has been reported that RORα can regulate normal physiological functions, such as lipid and steroid metabolism (12). An important aspect of RORα is that it has an anti-inflammatory role via inhibition of NF-κB (16–18, 45, 46). It was reported that RORα1 inhibits the expression of TNF-α-induced IL-6 and IL-8 via increasing IκBα in vascular smooth muscle cells and RORα1 and RORα4 reduces TNF-α-induced translocation of p50 and to the nucleus (16, 45). Furthermore, RORα significantly repressed the production of TNF-α in Kupffer cells (46). TNF-α-producing T cells are also known to play important pathogenic roles, but the effects of RORα on these T cells have not been elucidated yet. In this study, we identified that RORα could control the production of TNF-α in Th17 cells. A recent study indicated that RORα is a positive regulator of tumor suppressor p53, leading to increases in its transcriptional activity and protein stability (47). Interestingly, we found recently that p53 can alleviate autoimmune arthritis by regulating the balance between Th17 and Treg subsets through direct binding to STAT3 and STAT5 (22). Taken together, these observations suggest that RORα has anti-inflammatory functions in autoimmune diseases, such as RA.

RORγt, a member of the retinoic acid receptor-related orphan nuclear hormone receptor family, is expressed specifically under Th17 differentiation conditions (19). RORγt is a master transcription factor that drives Th17 cell lineage generation (19). Both RORγt and RORα are members of the retinoic acid receptor-related orphan nuclear hormone receptor family (48). Yang et al. demonstrated that RORα is also expressed by Th17 cells and that RORα overexpression can promote Th17 differentiation (20). They also reported that RORα and RORγt can synergistically lead to Th17 generation, suggesting that RORα plays a pivotal role in Th17 differentiation as another Th17 lineage-specific transcription factor. The present study indicated that RORα has anti-inflammatory effects and potentially antagonistic properties during Th17 differentiation. In addition, we found that overexpression of RORα activity in arthritic mice attenuated the expression of RORγt and IL-17. Cellular responses to any stimulus are context-dependent. We focused on the biological roles of RORα in autoimmune arthritis, which may explain, at least in part, the discrepancies between our results and those of Yang et al. in multiple sclerosis model (20). Multiple sclerosis is a disease in which T cells are primarily initiators and mediators whereas RA is a disease caused by the interaction of fibroblast-like synoviocytes with innate immune cells and adaptive immune cells. It was reported that RORα is capable of negative regulation in various cells including macrophages (18, 49). Furthermore, recently, it was reported that vitamin A can induce changes in gut microbiota composition and thereby increase the expression of CD38 and RORα (50). Considering these various possibilities, further studies are needed on the effect of RORα on T cells and other immune cells in RA.

Biologically, it is puzzling that another molecule, RORα, and RORγt have the same role in Th17 cells. Interestingly, Farez et al. recently reported that RORα activity can promote the generation of CD4^+^ IL-10-producing type 1 regulatory T cells in an animal model of experimental autoimmune encephalomyelitis, indicating the immunoregulatory potential of RORα (51). The regulation of effector T and Treg cells has been acknowledged mainly in the interaction with transcription factors. However, accumulating evidence suggests that changes in basic cellular metabolism also have an influence on T cell proliferation and cell fate. Activated T cells have to adjust their metabolic programs to satisfy the metabolic demands of biosynthetic precursors to have sufficient energy to participate in immune response (52–55). For example, activated CD4^+^ T cells are highly anabolic. They can increase glycolysis and increase glucose uptake to generate ATP and fundamental sources. In contrast, Tregs utilize lipid oxidation as a primary metabolic pathway to expand and function even in the absence of glucose (6, 56). Consistent with these findings, the results of the present study indicated that alterations of the glycolytic pathway following increased RORα activity may underlie the tendency toward Treg cell differentiation from naïve T cells rather than toward Th17 cells.

It is noteworthy that RORα appeared to regulate the differentiation of osteoclasts in RA. Bone tissue is a highly metabolically active and organized tissue, which is continuously remodeled to repair damage and maintain the balance through the concerted actions of bone cells, including bone resorption by osteoclasts and bone formation by osteoblasts. RORα has been implicated in the regulation of bone biology. RORα homozygous mutant mice have long thin bones and diminished total bone mineral content in the tibia compared to heterozygotes or wild-type mice (41). These observations suggest that RORα activity is related to osteoclastogenesis. Further studies are needed to understand the relationship between RORα and bone biology.

In conclusion, CS, a putative natural ligand of RORα, and SR1078, a selective RORα ligand, inhibited number of Th17 cells and IL-17 production. Fortification of RORα-mediated Th17 inhibition was associated with attenuated gene expression levels of glycolysis-associated molecules as well as p53. Overexpression of RORα reduced the clinical severity of arthritis and the extent of histological inflammation in a murine model of RA. In addition, RORα overexpression *in vivo* significantly attenuated the expression of proinflammatory cytokines, such as IL-17, IL-1β, IL-6, TNF-α, and VEGF. Furthermore, RORα activity inhibited osteoclastogenesis. These findings suggest that RORα may be a novel therapeutic target for RA management through inhibition of Th17 production and prevention of bone destruction.

## Data Availability Statement

All datasets generated for this study are included in the manuscript/supplementary files.

## Ethics Statement

All experimental procedures were examined and approved by the Animal Research Ethics Committee of the Catholic University of Korea, and conformed to the National Institutes of Health (USA) guidelines (permit number: 2014-0126-01, 2017-0139-03).

## Author Contributions

J-SP, S-JM, J-KM, S-HP, and M-LC: study design. J-SP, M-AL, J-KB, S-HH, SY, E-KK, HL, and S-MK: data acquisition. J-SP, S-JM, JL, S-KK, J-KM, M-OL, D-YS, S-HP, and M-LC: data analysis and interpretation. J-SP, S-JM, S-HP, and M-LC: manuscript drafting. All authors have critically reviewed the manuscript and approved the final manuscript.

### Conflict of Interest

The authors declare that the research was conducted in the absence of any commercial or financial relationships that could be construed as a potential conflict of interest.
